# Shock index and modified shock index are predictors of long-term mortality not only in STEMI but also in NSTEMI patients

**DOI:** 10.1080/07853890.2022.2056240

**Published:** 2022-04-04

**Authors:** Timo Schmitz, Eva Harmel, Jakob Linseisen, Inge Kirchberger, Margit Heier, Annette Peters, Christa Meisinger

**Affiliations:** a Chair of Epidemiology, University of Augsburg, University Hospital Augsburg, Augsburg, Germany; b Department of Cardiology, University Hospital of Augsburg, Augsburg, Germany; c IRG Clinical Epidemiology, Helmholtz Zentrum München, Munich Germany; d KORA Study Centre, University Hospital of Augsburg, Augsburg, Germany; e Institute of Epidemiology, Helmholtz Zentrum München, Munich Germany; f Chair of Epidemiology, Institute for Medical Information Processing, Biometry and Epidemiology, Medical Faculty, Ludwig-Maximilians-Universität München, Munich Germany; g German Center for Diabetes Research (DZD), Neuherberg, Germany

**Keywords:** Myocardial infarction, shock index, long-term mortality

## Abstract

**Background:**

Shock index (SI) and modified shock index (mSI) are useful instruments for early risk stratification in acute myocardial infarction (AMI) patients. They are strong predictors for short-term mortality. Nevertheless, the association between SI or mSI and long-term mortality in AMI patients has not yet been sufficiently examined.

**Material and methods:**

For this study, a total of 10,174 patients with AMI was included. All cases were prospectively recorded by the population-based Augsburg Myocardial Infarction Registry from 2000 until 2017. Endpoint was all-cause mortality with a median observational time of 6.5 years [IQR: 3.5–7.4]. Using ROC analysis and calculating Youden-Index, the sample was dichotomized into a low and a high SI and mSI group, respectively. Moreover, multivariable adjusted COX regression models were calculated. All analyses were performed for the total sample as well as for STEMI and NSTEMI cases separately.

**Results:**

Optimal cut-off values were 0.580 for SI and 0.852 for mSI (total sample). AUC values were 0.6382 (95% CI: 0.6223–0.6549) for SI and 0.6552 (95% CI: 0.6397–0.6713) for mSI. Fully adjusted COX regression models revealed significantly higher long-term mortality for patients with high SI and high mSI compared to patients with low indices (high SI HR: 1.42 [1.32–1.52], high mSI HR: 1.46 [1.36–1.57]). Furthermore, the predictive ability was slightly better for mSI compared to SI and more reliable in NSTEMI cases compared to STEMI cases (for SI and mSI).

**Conclusion:**

High SI and mSI are useful tools for early risk stratification including long-term outcome especially in NSTEMI cases, which can help physicians to make decision on therapy. NSTEMI patients with high SI and mSI might especially benefit from immediate invasive therapy.

**Key messages**
- Shock index and modified shock index are predictors of long-term mortality after acute myocardial infarction.
- Both indices predict long-term mortality not only for STEMI cases, but even more so for NSTEMI cases.

## 1. Introduction

The shock index (SI), defined as heart rate divided by systolic blood pressure, was first mentioned in 1967 as an additional tool for the evaluation of the hemodynamic stability of patients [1]. Since then, many studies examined whether the SI as well as the modified SI (mSI), defined as heart rate divided by mean arterial blood pressure, are useful tools for early risk stratification in patients with different underlying diseases in the emergency department [2]. Among the examined diseases and conditions were traumatic injuries, sepsis, ectopic pregnancy in obstetrics or cardiovascular diseases such as pulmonary embolism [2]. In patients with acute myocardial infarction (AMI), several prior studies demonstrated that both, SI and mSI, are reliable predictors of short-term outcomes [3–6]. Since SI and mSI can be easily determined at the very beginning of emergency treatment, they become useful instruments for early risk stratification in AMI patients.

Several studies further indicated that SI is also associated with mid- and even long-term mortality after AMI [4,7–12]. Nevertheless, most of these studies either lack a longer follow-up period (more than 1 year), or a high number of included cases (more than 1,000 included patients). Furthermore, most studies only included cases of ST-elevation myocardial infarction (STEMI) events and only two studies also investigated non-ST-elevation myocardial infarction (NSTEMI) cases [7,13]. To the best of our knowledge, no prior studies based on real-world data from population-based registries have been conducted so far.

Thus, the objective of this study was to further examine the association between SI and mSI and all-cause long-term mortality in AMI patients. The study is based on data from a population-based registry with consecutive enrolment over a long period of time. It is characterized by a high number of cases and a long follow-up period. Furthermore, we tried to investigate whether SI and mSI are not only predictors of long-term mortality in STEMI cases, but in NSTEMI cases as well.

## 2. Material and methods

### 2.1. Patients

The underlying data for this research were collected by the population based Augsburg Myocardial Infarction Registry. It was established in 1984 as a part of the MONICA-project (Monitoring Trends and Determinants in Cardiovascular disease) and since then operated as KORA Myocardial Infarction Registry [14]. The study area consists of the city of Augsburg, Germany, and the two adjacent counties comprising a total of approximately 680,000 inhabitants. For this analysis, all cases of hospitalized AMI were recorded on the following conditions: patients age was between 25 and 74 years (2000 until 2008) or between 25 and 84 years (2009 until 2017), the patient survived the first 24 h after hospital admission and had its primary residence within the study area. Trained study nurses carried out interviews using standardized questionnaires during the hospital stay. Furthermore, clinical data were collected by medical chart review. In this way, a large amount of data for each case of AMI was collected including sociodemographic characteristics, risk factors, comorbidities, diagnostics and treatment. Mortality follow-ups were performed regularly in order to keep data on long-term survival of patients up to date. Therefore, necessary information was obtained from the regional registration offices and health offices. For this study, the last extensive mortality follow-up update was performed in 2019. More detailed information on data collection is available in previous publications [15,16]. Data collection of this registry has been approved by the ethics committee of the Bavarian Medical Association (Bayerische Landesärztekammer) and the study was performed in accordance with the Declaration of Helsinki. All study participants have given written informed consent.

Since the present study aimed to investigate long-term survival exclusively, patients who died within the first 28 days after AMI (short-term mortality) were excluded.

The variable “shock index” (SI) was defined as heart rate [bpm] divided by systolic blood pressure (SBP) in mmHg (SI = HR/SBP).

The variable “modified shock index” (mSI) used mean arterial pressure (MAP), which also includes diastolic blood pressure in mmHg (DBP), instead of solely SBP. MAP is defined as the following: MAP = (2 × DBP + SBP)/3. Consequently; the formula for mSI was: mSI = HR/MAP.

By performing ROC analyses and calculating the Youden-Index for 3-year all-cause mortality (for detailed description see below), optimal cut-off values were determined separately for SI and mSI and for the total sample as well as separately for the STEMI and NSTEMI samples. According to those cut-off values, all cases were assigned to either the low group (patients with low SI/low mSI) or the high group (patients with high SI/high mSI).

Admission ECG was evaluated by clinical physicians. Each case was assigned to either the STEMI group or the NSTEMI group.

Estimated GFR was calculated by admission creatinine levels according to the CKD-EPI formula. Four categories were defined: normal renal function (eGFR >60 ml/min/1.73 m^2^), slightly impaired renal function (eGFR between 30 and 60 ml/min/1.73 m^2^), heavily impaired renal (eGFR <30 ml/min/1.73 m^2^) and no information on renal function (values for creatinine levels were only available since 2005).

For left-ventricular ejection fraction (EF), three categories were built: “severely reduced left-ventricular EF” (≤30%), “not severely reduced EF” (>30%) and “no-information on left-ventricular EF”.

For any in-hospital complication including cardiogenic shock, left ventricular decompensation, bradycardia, reinfarction, ventricular tachycardia and ventricular fibrillation, one variable was generated (yes/no).

Regarding acute treatment, it was recorded whether the following methods were conducted within the hospital stay after the AMI event: percutaneous coronary intervention (PCI), coronary bypass graft surgery, thrombolysis (yes/no).

### 2.2. Statistical analysis

Baseline characteristics are presented as total numbers and percentages for categorical variables mean and standard deviation (SD) for continuous variables. To determine differences in baseline characteristics between the shock groups, Chi^2^ test for categorical variables and one-way ANOVA (analysis of variance) for continuous variables were performed.

As the subsequent statistical analyses were performed for the total sample (including STEMI and NSTEMI cases) as well as for both groups separately, the baseline characteristics stratified for STEMI and NSTEMI are provided in the Supplementary material.

#### ROC analysis

For SI and mSI, we intended to find optimal cut-off values for the analysis on long-term mortality. Therefore, ROC curves (receiver operating characteristic) and AUC (area under the curve) were calculated. As this requires dichotomous outcome variables, we have chosen 3-year survival as outcome variable. All cases with missing information on 3-year survival were removed for the ROC analysis. For the subsequent COX regression analyses, all cases were included. To find the optimal cut-off values, the Youden index (= sensitivity + specificity − 1) was calculated. As cut-off points SI and mSI values with maximal Youden index were chosen. These calculations were performed for the total sample including all AMI cases and for STEMI and NSTEMI cases separately. To compare the goodness of discrimination between SI and mSI, bootstrapping methods were used to calculate *p*-values for differences in AUC (number of replicates: 2,000). Furthermore, we calculated IDI (integrated discrimination increment) and continuous NRI (continuous net reclassification index) to compare both indices with regards to their predictive ability.

**Figure 1.**
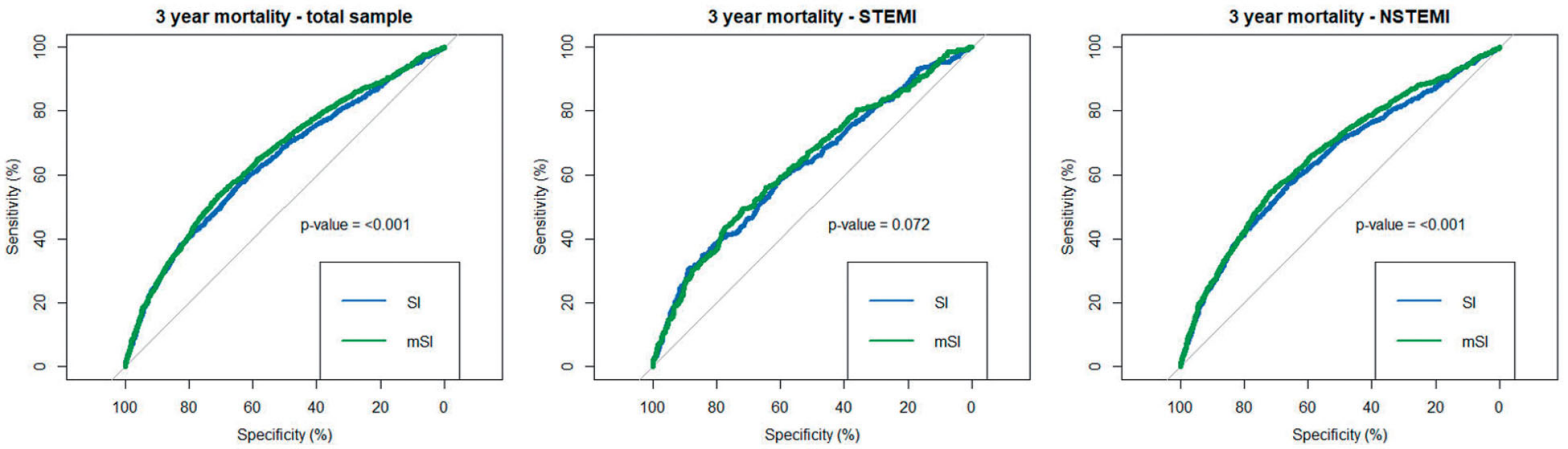
ROC curves for 3-year mortality (total sample, STEMI cases, NSTEMI cases). *p*-values are calculated by comparing the AUC between SI and mSI using bootstrapping.

**Figure 2.**
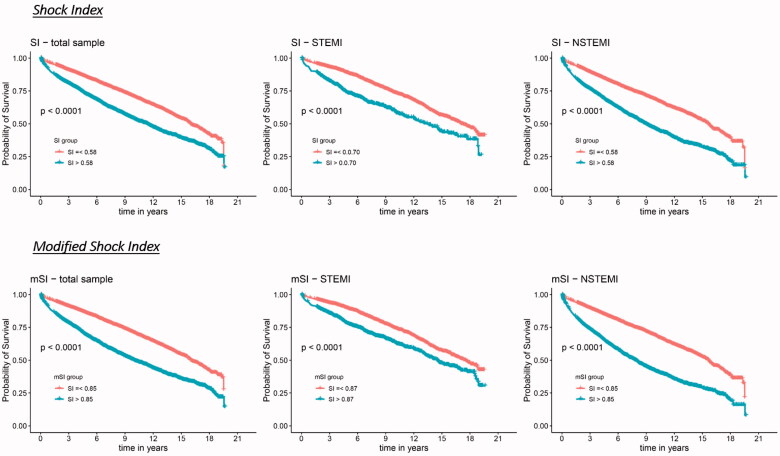
Kaplan–Meier survival curves by shock index groups and modified shock index groups respectively for the total sample, STEMI and NSTEMI.

In the Supplementary material, we also display the results and graphs of the 3-years survival ROC analysis calculated with all patients including those who died within the first 28 days after the event (Figure 1 and Tables 4 and 6). Furthermore, we performed the same analyses also for 28 day mortality including all patients (Figure 2 and Tables 5 and 6).

#### COX regression analysis

To investigate the association between SI and long-term mortality, three different COX regression models were calculated. The first model included only the categorized variable “shock index” or “modified shock index,” respectively. The second model was adjusted for sex and age. The final model was further adjusted for a variety of relevant covariates and potential confounders. In order to avoid overfitting, the number of covariables was restricted to a maximum of 15. The following covariables were included into the final model: sex, age, typical chest pain symptoms, diabetes, hyperlipidaemia, hypertension, smoking status, left-ventricular EF (≤30%), impaired renal function (categorized according to GFR), PCI, bypass surgery, thrombolysis therapy and any in-hospital complication.

The proportional hazards assumption was checked by plotting the Schoenfeld residuals against time and searching for any visible correlation. Additionally, a test was performed to check for a significant correlation of the Schoenfeld residuals with time and consequently a violation of the proportional hazards assumption. Furthermore, log(−log(Survival)) plots were inspected visually for crossing curves. Since many covariables violated the proportional hazards assumption – most likely as a consequence of the long follow-up period – a time step function was implemented for all covariables (but not for the SI and mSI variable) in the parsimonious model (time split at 2,500 days after AMI).

For the variables of interest, SI and mSI, which also violated the proportional hazards assumption, a time split function was implemented as well (time splits: after 1, 3, 6, 9 and 12 years). The results for these models are displayed in the Supplementary material (Table 3).

Just like the calculations for the optimal cut-off points, the calculations for the COX models were performed for the total sample as well as for STEMI and NSTEMI cases separately.

The statistical analysis was performed by R version 3.6.1.

## 3. Results

### 3.1. Baseline characteristics

Patients’ baseline characteristics are summarized in Table 1. The number of women was significantly higher in the groups with high SI and high mSI. Mean age, however, was not different between groups. The groups with higher SI and mSI were significantly longer treated in intensive care units and had significantly more often in-hospital complications. Moreover, peak CKMB and peak CRP levels were significantly higher in these groups. In regard to treatment, the frequency of PCI was higher in patients with low SI and low mSI, while bypass therapy was more commonly performed in the patients with high shock indices. Further differences in major baseline characteristics are displayed in Table 1.

**Table 1.** Baseline characteristics of patients with available data on long-term survival (total sample).

	Shock index (SI)				Modified shock index (mSI)			
	SI ≤ 0.58 (*n* = 6250)	SI >0.58 (*n* = 3924)	*p*-value	n	mSI ≤ 0.85 (*n* = 6917)	mSI >0.85 (*n* = 3257)	*p*-value	*n*
Total number	6250 (61.4)	3924 (38.6)	–	10,174	6917 (68.0)	3257 (32.0)	–	10,174
Sex (male)	4674 (74.8)	2749 (70.1)	<.001	10,174	5196 (75.1)	2227 (68.4)	<.001	10,174
Age	63.8 (10.9)	63.8 (11.5)	.8305	10,174	63.4 (10.9)	64.6 (11.4)	<.001	10,174
Number of patients who survived >3 years (survival rate)	5172 (90.3)	2884 (79.6)	<.001	9350	5719 (90.4)	2337 (77.2)	<.001	9350
*Vital parameters*								
Heart rate	71.4 (12.8)	94.9 (20.7)	<.001	10,174	72.5 (13.2)	97.4 (21.2)	<.001	10,174
Systolic blood pressure	155.7 (24.8)	129.2 (23.8)	<.001	10,174	153.2 (25.3)	129.2 (25.2)	<.001	10,174
Diastolic blood pressure	85.8 (15.9)	77.2 (16.1)	<.001	10,174	86.7 (15.4)	73.6 (15.3)	<.001	10,174
SI	0.46 (0.07)	0.75 (0.19)	<.001	10,174	0.48 (0.09)	0.77 (0.20)	<.001	10,174
mSI	0.66(0.11)	1.03 (0.26)	<.001	10,174	0.67 (0.1)	1.07 (0.26)	<.001	10,174
*Comorbidities*								
Hypertension	5050 (80.8)	2913 (74.2)	<.001	10,174	5510 (79.7)	2453 (75.3)	<.001	10,174
Diabetes	1855 (29.7)	1369 (34.9)	<.001	10,174	2018 (29.2)	1206 (37)	<.001	10,174
Hyperlipidaemia	4039 (64.6)	2317 (59)	<.001	10,174	4439 (64.2)	1917 (58.9)	<.001	10,174
Smoking status								
Current smoker	1889 (30.2)	1354 (34.5)	<.001	10,174	2179 (31.5)	1064 (32.7)	<.001	10,174
Never smoker	1996 (31.9)	1190 (30.3)	–	10,174	2179 (31.5)	1007 (30.9)	–	10,174
Ex-smoker	2051 (32.8)	1056 (26.9)	–	10,174	2219 (32.1)	888 (27.3)	–	10,174
No information	314 (5)	324 (8.3)	–	10,174	340 (4.9)	298 (9.1)	–	10,174
*Clinical characteristics*								
Typical chest-pain symptoms	5442 (87.1)	2935 (74.8)	<.001	10,174	6018 (87)	2359 (72.4)	<.001	10,174
Heart rhythm (admission ECG)								
Sinus rhythm	1652 (93)	958 (82.7)	<.001	2935	1820 (92.7)	790 (81.4)	<.001	2935
Atrial fibrillation	99 (5.6)	166 (14.3)	–		117 (6)	148 (15.2)	–	2935
Pacemaker rhythm	4 (0.2)	1 (0.1)	–		4 (0.2)	1 (0.1)	–	2935
Ventricular tachycardia/ventricular fibrillation	1 (0.1)	5 (0.4)	–		1 (0.1)	5 (0.5)	–	2935
Other type/unknown	21 (1.2)	28 (2.4)	–		22 (1.2)	27 (2.8)	–	
*Type of infarction*								
STEMI	2225 (35.6)	1512 (38.5)	<.001	10,174	2532 (36.6)	1205 (37)	<.001	10,174
NSTEMI	3598 (57.6)	2052 (52.3)	–	10,174	3922 (56.7)	1728 (53.1)	–	10,174
BBB	427 (6.8)	360 (9.2)	–	10,174	463 (6.7)	324 (9.9)	–	10,174
Days in intensive care unit	2.7 (4)	4.3 (6.9)	<.001	9865	2.7 (4.2)	4.5 (7.1)	<.001	9865
Any in-hospital complication^a^	828 (13.2)	731 (18.6)	<.001	10,174	909 (13.1)	650 (20)	<.001	10,174
In-hospital complication: cardiogenic shock	85 (1.4)	219 (5.6)	<.001	10,174	99 (1.4)	205 (6.3)	<.001	10,174
*Left ventricular EF*								
≤30%	152 (2.4)	359 (9.1)	<.001	10,174	186 (2.7)	325 (10)	<.001	10,174
>30%	4832 (77.3)	2697 (68.7)	–	10,174	5346 (77.3)	2183 (67)	–	10,174
No information on EF	1266 (20.3)	868 (22.1)	–	10,174	1385 (20)	749 (23)	–	10,174
*Kidney function*								
eGFR >60 (ml/min/1.73 m²)	3602 (57.6)	1841 (46.9)	<.001	10,174	3997 (57.8)	1446 (44.4)	<.001	10,174
eGFR 30-60 (ml/min/1.73 m²)	1011 (16.2)	921 (23.5)	–	10,174	1070 (15.5)	862 (26.5)	–	10,174
eGFR <30 (ml/min/1.73 m²)	170 (2.7)	227 (5.8)	–	10,174	190 (2.7)	207 (6.4)	–	10,174
Missing information on eGFR	1467 (23.5)	935 (23.8)	–	10,174	1660 (24)	742 (22.8)	–	10,174
Peak CK-MB (U/l)	98.7 (124)	115.1 (161.9)	<.001	8999	101.2 (126.9)	113.2 (164.5)	<.001	8999
Peak CRP levels (mg/l)	6.4 (8)	9.7 (9.4)	<.001	9867	6.5 (8.1)	10.1 (9.5)	<.001	9867
*Treatment*								
PCI	4686 (75)	2470 (62.9)	<.001	10,174	5178 (74.9)	1978 (60.7)	<.001	10,174
Bypass therapy	849 (13.6)	630 (16.1)	<.001	10,174	953 (13.8)	526 (16.1)	.0017	10,174
i.v. thrombolysis therapy	261 (4.2)	164 (4.2)	1	10,174	302 (4.4)	123 (3.8)	.1823	10,174
Any reperfusion therapy	5500 (88)	3090 (78.7)	<.001	10,174	6092 (88.1)	2498 (76.7)	<.001	10,174
*Medication at discharge*								
ACE blockers	4675 (76.7)	2828 (74.4)	.0084	9897	5195 (77)	2308 (73.3)	<.001	9897
ATII antagonist	610 (10)	333 (8.8)	.0428	9894	650 (9.6)	293 (9.3)	.6408	9894
Beta blockers	5761 (94.5)	3591 (94.4)	.7693	9899	6397 (94.8)	2955 (93.8)	.0656	9899
Antiplatelet drug	5993 (98.3)	3633 (95.5)	<.001	9899	6625 (98.1)	3001 (95.3)	<.001	9899
Statins	5596 (91.8)	3331 (87.6)	<.001	9897	6201 (91.9)	2726 (86.6)	<.001	9897

Categorical data are presented as total numbers (%). Numeric data are presented as mean (SD).

^a^Including cardiogenic shock, left ventricular decompensation, bradycardia, reinfarction, ventricular tachycardia and ventricular fibrillation.

### 3.2. Roc analysis

Figure 1 displays the ROC curves for 3-year mortality. The AUC for the total sample was 0.6382 (95% CI: 0.6223–0.6549) for SI and 0.6552 (95% CI: 0.6397–0.6713) for mSI (*p*-value: <.001). For STEMI cases, there were no significant differences between SI and mSI with AUC values of 0.6250 (95% CI: 0.5907–0.6576) for SI and 0.6338 (95% CI: 0.5987–0.6665) for mSI (*p*-value: .072). For NSTEMI cases, the AUC values were 0.6453 (95% CI: 0.6252–0.6646) for SI and 0.6631 (95% CI: 0.6453–0.682) for mSI (*p*-value: <.001). Overall, the AUC values indicated a more reliable prediction of 3-year mortality for NSTEMI cases than for STEMI cases, especially for mSI. Yet, statistical comparison revealed no significant difference (SI *p*-value: .3034, mSI *p*-value: .2932).

Optimal cut-off values were identified by maximizing Youden-Index, which revealed the following cut-off values: for the *total sample*: SI: 0.5803226 (Youden-Index: 0.215186), mSI: 0.8524522 (Youden-Index: 0.243678); for *STEMI*: SI: 0.702591 (Youden-Index: 0.193344), mSI: 0.87000 (Youden-Index: 0.210521); for *NSTEMI*: SI: 0.5803226 (Youden-Index: 0.231327), mSI: 0.8524522 (Youden-Index: 0.264642).

For the total sample, the comparison of SI versus mSI revealed a IDI of 0.006 (95%CI: 0.004–0.008, *p*-value: <.001) and a continuous NRI of 0.29 (95%CI: 0.233–0.347, *p*-value: <.001). For STEMI cases, the IDI was 0.001 (95%CI: −0.001 to 0.002, *p*-value: .394) and the continuous NRI was 0.075 (95%CI: −0.039 to 0.189, *p*-value: .197). For NSTEMI cases on the other hand, there was a greater difference between SI and mSI: IDI: 0.008 (95%CI: 0.006–0.010, *p*-value: <.001) and continuous NRI: 0.309 (95%CI: 0.244–0.375, *p*-value: <.001).

### 3.3. Cox regression analysis

To further investigate the association between SI and mSI and long-term mortality, COX regression models were calculated. After excluding all cases with missing values for relevant covariables as well as missing information on long-term survival, 10,174 patients were taken into account for the final analysis. The median follow-up time was 6.5 years (IQR 3.5–10.6). During the follow-up period, 3522 patients died (34.6%).

Figure 2 displays the unadjusted survival curves (Kaplan–Meier curves) stratified by SI and mSI for the total sample and STEMI and NSTEMI cases separately. As the ROC analyses indicated, Kaplan–Meier curves also revealed a better discrimination in the NSTEMI group compared to the STEMI group for both SI and mSI (in contrast to the ROC analysis, which has taken into account only the 3-year survival, the Kaplan–Meier curves considered the total observational time).

The summarized results of the COX regression models can be found in Table 2. The “low group” was set as the reference group in each of the models. The unadjusted model revealed a significantly higher mortality for the patients with high SI and mSI (in the total sample as well as in STEMI and NSTEMI cases). After adjusting for sex and age, the relative risks even increased slightly. In the fully adjusted model, the Hazard ratios decreased noticeably, but remained significant for SI and mSI and for all samples. Overall, HR values were slightly higher for mSI compared to SI, and in the NSTEMI group compared to the STEMI group.

**Table 2.** Results of the COX regression models for SI and mSI with all cause-mortality as end-point.

	Shock index		Modified shock index	
Shock group	HR [95% CI]	*p*-value	HR [95% CI]	*p*-value
*Unadjusted model*				
Total sample				
~ Low	1	–	1	–
~ High	1.74 [1.63–1.86]	<.001	1.97 [1.85–2.11]	<.001
STEMI				
~ Low	1	–	1	–
~ High	1.41 [1.25–1.59]	<.001	1.53 [1.35–1.73]	<.001
NSTEMI				
~ Low	1	–	1	–
~ High	1.97 [1.82–2.13]	<.001	2.25 [2.07–2.43]	<.001
*Adjusted for sex and age*				
Total sample				
~ Low	1	–	1	–
~ High	1.85 [1.73–1.98]	<.001	1.97 [1.84–2.10]	<.001
STEMI				
~ Low	1	–	1	–
~ High	1.53 [1.36–1.73]	<.001	1.55 [1.37–1.76]	<.001
NSTEMI				
~ Low	1	–	1	–
~ High	2.05 [1.89–2.22]	<.001	2.22 [2.05–2.40]	<.001
*Fully adjusted^a^*				
Total sample				
~ Low	1	–	1	–
~ High	1.42 [1.32–1.52]	<.001	1.46 [1.36–1.57]	<.001
STEMI				
~ Low	1	–	1	–
~ High	1.25 [1.10–1.42]	<.001	1.27 [1.12–1.45]	<.001
NSTEMI				
~ Low	1	–	1	–
~ High	1.52 [1.39–1.65]	<.001	1.59 [1.46–1.73]	<.001

^a^Adjusted for sex, age, typical chest pain symptoms, diabetes, smoking, hyperlipidaemia, hypertension, left-ventricular EF ≤ 30%, impaired renal function (according to GFR), any in-hospital complication, PCI, Bypass surgery and Lysis therapy.

Since the SI and mSI variable violated the proportional hazards assumption, the same COX model with time split functions was calculated (see Supplementary material). It revealed that the Hazard ratios are higher in the earlier time periods and attenuated for the latter ones.

## 4. Discussion

In this study, we investigated the association between SI and mSI and all-cause long-term mortality after AMI. According to the optimal cut-off values determined by Youden-Index, about one-third of all patients have been assigned to the high SI and mSI group (38.6% for SI and 32.0% for mSI, respectively). Comparison of baseline characteristics revealed some major differences between the high and low groups. Overall, it appears that patients assigned to the high groups had more severe infarctions than those in the low groups: longer mean duration in intensive care units, higher prevalence of in-hospital complications, higher peak-CKMB and peak CRP levels, more often performed bypass therapy. Especially the higher peak CK-MB levels suggest higher myocardial damage in the groups with high shock indices [17,18].

In a 3-years survival ROC analysis, AUC values ranged between 0.6 and 0.7 with the lowest value for SI in STEMI patients (AUC: 0.6382) and the highest for mSI in NSTEMI cases (AUC: 0.6631). Prior studies which performed ROC analysis for SI and/or mSI on prediction of mid- to long-term survival after AMI came to similar results with AUC values ranging between 0.6 and 0.7 [4,5,8,12].

To further investigate the association between SI and mSI and long-term all-cause mortality and in order to take potential confounders into account, we conducted COX-regression models. The fully adjusted model revealed a significantly higher mortality for the high SI and mSI groups for both, the total sample and the divided STEMI/NSTEMI groups. It was found that the predictive value attenuated over time, but the trend of a higher mortality for the high groups remained significant even in the latter time periods. For a group of 1,369 STEMI cases, Ndrepepa et al. reported that high SI was associated with higher long-term mortality up to 8 years, nevertheless this adverse long-term prognosis was almost entirely driven by deaths within the first 30 days after the event [10]. That would imply that the effect of higher mortality is actually restricted to a short period of time after the event. In our study we excluded patients who died within the first 28 days after the event in order to purely concentrate on long-term mortality. And despite doing so, high SI and mSI were strongly associated with higher mortality even in the long term.

The points discussed above are true not only for the total sample of AMIs (STEMI and NSTEMI), but they also remain valid when looking at STEMI and NSTEMI cases separately. Though, the overall prediction of long-term mortality by SI and mSI is more accurately for NSTEMI cases than for STEMI cases. This has rarely been investigated in prior studies and so we cannot compare our results properly to previous studies. At this point, one can only speculate about the reasons for this circumstance. First to mention is the assumption of STEMI cases being much more homogeneous in terms of patients’ characteristics, disease severity and acute treatment. Therein, the NSTEMI group is much more heterogeneous and the influence of different underlying diseases and comorbidities (like severe diabetes mellitus) as well as differences in treatment are much greater. So, SI and mSI might be highly correlated with the overall health condition of a patient, which might be much more diverse in the NSTEMI group. This circumstance perhaps accounts for the better distinction in regard to long-term mortality in NSTEMI cases as compared to STEMI cases.

The final COX models in this study were adjusted for sex and age, typical comorbidities and risk factors as well as important parameters on treatment. This eliminated the prediction mediated by these variables and allowed estimation of the independent predictive value of SI and mSI. As expected, adjusting the model reduced the HR values of the high SI and the high mSI group. Nevertheless, high SI and mSI remained significantly associated with a higher long-term mortality, which indicates that the predictive power of SI and mSI is partially mediated by the overall health status of the patients but also partly independent of it. In this regard of course, one must be aware of residual confounding.

Finally, we compared both indices, SI and mSI, according to their power of discrimination. Prior studies indicated, that mSI might have a greater accuracy for the prediction of adverse outcomes in different diseases and conditions [19–21]. For example, a large study including over 22,000 patients from Liu et al. suggested, that in a general group of emergency patients mSI might be superior to SI in predicting mortality [22]. In the present study, AUC values for mSI were significantly higher than for SI in the total sample and in NSTEMI cases, however the graphs appear to be very comparable and no conspicuous superiority of the mSI could be noticed. In subsequent analysis we calculated IDI and continuous NRI. First of all, there was no significant difference between SI and mSI among STEMI patients. This results confirmed a study by Reinstadler et al., as they found no significant differences between SI and mSI for predicting 12 months adverse outcome (including death) in a sample of 791 STEMI patients [4]. Our results suggest that the predictive values of SI and mSI remain comparable even many years after the acute event in STEMI cases.

For NSTEMI patients on the other hand, the situation appears to be different. In the present study, IDI and continuous NRI suggested a significant, but rather moderate superiority of mSI over SI. In a single-centre study from China, Yu et al. compared the predictive performance of SI and mSI in 1864 AMI patients regarding long-term outcome (mean follow-up time: 32 months) [5]. They reported a significant, but also moderate superiority of mSI compared to SI (NRI: 0.346, IDI: 0.002). However, the study sample was a combined sample of STEMI an NSTEMI cases (in equal shares), which does not allow to draw a specific conclusion on NSTEMI cases. The presents study would suspect that this superiority is mainly driven by the NSTEMI cases and rather not so by STEMI cases.

Now there remains the question whether these differences for mSI compared to SI are relevant in a clinical setting. The question can easily be answered with no for STEMI cases. For NSTEMI cases on the other hand, it appears to be beneficial for clinicians to use mSI instead if SI. It is pivotal for any early risk stratification parameter in clinical practice to be kept simple and easy to determine. For both, calculation of SI and mSI, blood pressure and heart rate values are required. Since calculation for SI is very simple, it can be immediately estimated trough mental arithmetic by every physician. The calculation of mSI on the other hand is a bit more challenging and not quite as intuitive. Admittedly, this is not really an issue in a setting of modern medicine diagnostics (MAP is standardly calculated by any blood pressure monitor nowadays). Nevertheless, there might be some cases, e.g. in a prehospital environment, where this point becomes relevant.

## 5. Strengths and limitations

This study is characterized by some strengths. First to mention is the high number of included cases from a population-based registry with consecutive enrolment, which reduces the risk of selection bias. Moreover, not only STEMI cases but also NSTEMI-type events were included in this study, and the statistical analysis was performed for the total sample of AMI as well as for STEMI and NSTEMI cases separately, which allows to make statements for the subgroups (especially NSTEMI cases) as well. Furthermore, the post-event observation period was very long (median follow-up time of 6.5 years). In addition to information on the actual event, a large number of sociodemographic data, risk factors, comorbidities and information on in-hospital complications and treatment were collected for each case, which enabled multivariable adjustment.

Nevertheless, there are some limitations to our study as well. First of all, we had no information on cause of death (cardiovascular versus non-cardiovascular) and consequently we were not able to perform a subgroup analysis in this regard. Second, there was no information on certain cardiovascular drugs like catecholamines which strongly affect blood pressure and consequently SI or mSI. Since only patients up to 74 years (2000 until 2008) and up to 85 years (2009 until 2017) were included, results cannot be applied to older patients. In the almost two decades of case recording (18 years), processes and standards in diagnostics and treatment of AMI patients have changed considerably, which might have affected the results. Furthermore, our findings may not be generalized to all ethnic groups since no information on ethnicity was available. Moreover, we might not have considered all relevant confounders.

## 6. Conclusion

With regards to long-term mortality, SI and mSI are useful tools for early risk stratification not only in STEMI cases, but even more in NSTEMI cases, for which prediction appears to be more accurately than for the STEMI group. Beyond that, high SI and mSI are furthermore independently associated with higher long-term mortality. Based on these results physicians can be encouraged to take SI and mSI into account for early risk stratification. Especially in NSTEMI cases, SI and mSI help to identify high risk patients who might particularly benefit from immediate invasive treatment.

## Supplementary Material

Supplemental Material

## Data Availability

The data will not be shared. Due to restrictions from Helmholtz Zentrum München, data are available upon request for any researcher based on a standard agreement on data provision within the KORA Research Platform.
