# Supplementary material for: Shock index and modified shock index are predictors of long-term mortality not only in STEMI but also in NSTEMI patients
Source: Ann Med. 2022 Apr 4;54(1):900–8. doi: 10.1080/07853890.2022.2056240 (PMC8986179; doi:10.1080/07853890.2022.2056240)
Supplement: Supplemental Material [file IANN_A_2056240_SM7081.docx]

***Supplementary material***

***Shock index and modified shock index are predictors of long-term mortality not only in STEMI but also in NSTEMI patients.***

***Table 1:*** *Baseline characteristics of STEMI patients with available data on long-term survival. Categorical data presented as total numbers (%). Numeric data is presented as mean (SD).*

| ***STEMI*** | ***Shock Index*** | | | | ***Modified shock index*** | | | |
| --- | --- | --- | --- | --- | --- | --- | --- | --- |
|  | ***SI ≤ 0.70 (n=3080)*** | ***SI > 0.70 (n=657)*** | ***p-value*** | ***n*** | ***mSI ≤ 0.87 (n=2116)*** | ***mSI > 0.87 (n=1121)*** | ***p-value*** | ***n*** |
| ***sex male*** | *2323 (75.4)* | *450 (68.5)* | *< 0.001* | *3737* | *1980 (75.7)* | *793 (70.7)* | *0.0018* | *3737* |
| ***age*** | *61.6 (11.2)* | *61 (12)* | *0.2237* | *3737* | *61.4 (11.1)* | *61.7 (11.8)* | *0.5736* | *3737* |
| ***Number of patients who survived > 3 years (survival rate)*** | *2633 (92.6)* | *487 (81.3)* | *< 0.001* | *3442* | *2240 (93.2)* | *880 (84.8)* | *< 0.001* | *2704* |
| ***Vital parameters*** | | | | | | | | |
| ***Heart rate*** | *74.9 (15)* | *99.6 (20.2)* | *< 0.001* | *3737* | *72.6 (13.9)* | *94.8 (18.9)* | *< 0.001* | *3737* |
| ***Systolic blood pressure*** | *149 (26.1)* | *117.1 (23.2)* | *< 0.001* | *3737* | *150.9 (26.2)* | *125.8 (25.3)* | *< 0.001* | *3737* |
| ***Diastolic blood pressure*** | *84.9 (16.8)* | *72.6 (16.7)* | *< 0.001* | *3737* | *87.2 (16.3)* | *72.3 (15.4)* | *< 0.001* | *3737* |
| ***SI*** | *0.5 (0.1)* | *0.9 (0.2)* | *< 0.001* | *3737* | *0.5 (0.1)* | *0.8 (0.2)* | *< 0.001* | *3737* |
| ***mSI*** | *0.7 (0.1)* | *1.2 (0.3)* | *< 0.001* | *3727* | *0.7 (0.1)* | *1.1 (0.2)* | *< 0.001* | *3737* |
| ***Comorbidities*** | | | | | | | | |
| ***hypertension*** | *2300 (74.7)* | *417 (63.5)* | *< 0.001* | *3737* | *1967 (75.2)* | *750 (66.9)* | *< 0.001* | *3737* |
| ***diabetes*** | *843 (27.4)* | *193 (29.4)* | *0.3199* | *3737* | *697 (26.6)* | *339 (30.2)* | *0.027* | *3737* |
| ***hyperlipidemia*** | *1899 (61.7)* | *360 (54.8)* | *0.0013* | *3737* | *1626 (62.2)* | *633 (56.5)* | *0.0013* | *3737* |
| ***Smoking status*** | | | | | | | | |
| *current smoker* | *1178 (38.2)* | *286 (43.5)* | *< 0.001* | *3737* | *1001 (38.3)* | *463 (41.3)* | *< 0.001* | *3737* |
| *never smoker* | *859 (27.9)* | *156 (23.7)* | *-* |  | *727 (27.8)* | *288 (25.7)* | *-* |  |
| *ex-smoker* | *937 (30.4)* | *159 (24.2)* | *-* |  | *801 (30.6)* | *295 (26.3)* | *-* |  |
| *no information on smoking status* | *106 (3.4)* | *56 (8.5)* | *-* |  | *87 (3.3)* | *75 (6.7)* | *-* |  |
| ***Clinical characteristics*** | | | | | | | | |
| ***typical chest-pain symptoms*** | *2793 (90.7)* | *510 (77.6)* | *< 0.001* | *3737* | *2409 (92.1)* | *894 (79.8)* | *< 0.001* | *3737* |
| ***Heart rhythm (admission ECG)*** | | | | | | | | |
| *Sinus rhythm* | *830 (94.3)* | *157 (84.9)* | *0.0235* | *1065* | *706 (94.1)* | *281 (89.2)* | *0.0436* | *1065* |
| *atrial fibrillation* | *39 (4.4)* | *23 (12.4)* | *-* |  | *35 (4.7)* | *27 (8.6)* | *-* |  |
| *pacemaker rhythm* | *0 (0)* | *0 (0)* | *-* |  | *0 (0)* | *0 (0)* | *-* |  |
| *ventricular tachycardia/ ventricular fibrillation* | *0 (0)* | *1 (0.5)* | *-* |  | *0 (0)* | *1 (0.3)* | *-* |  |
| *other type / unknown* | *11 (1.3)* | *4 (2.2)* | *-* |  | *9 (1.2)* | *6 (1.9)* | *-* |  |
| ***days in intensive care*** | *2.7 (3.9)* | *5.1 (8.4)* | *< 0.001* | *3696* | *2.6 (3.7)* | *4.3 (7.1)* | *< 0.001* | *3696* |
| ***Any in-hospital complication*** | *590 (19.2)* | *193 (29.4)* | *0.0035* | *3737* | *484 (18.5)* | *299 (26.7)* | *< 0.001* | *3737* |
| ***In-hospital complication: cardiogenic shock*** | *82 (2.7)* | *81 (12.3)* | *< 0.001* | *3737* | *59 (2.3)* | *104 (9.3)* | *< 0.001* | *3737* |
| ***left ventricular EF*** | | | | | | | | |
| *≤ 30%* | *112 (3.6)* | *93 (14.2)* | *< 0.001* | *3737* | *83 (3.2)* | *122 (10.9)* | *< 0.001* | *3737* |
| *> 30%* | *2415 (78.4)* | *448 (68.2)* | *-* |  | *2073 (79.2)* | *790 (70.5)* | *-* |  |
| *no information on EF* | *553 (18)* | *116 (17.7)* | *-* |  | *460 (17.6)* | *209 (18.6)* | *-* |  |
| ***Kidney function*** | | | | | | | | |
| *eGFR > 60 (ml/min/1.73m²)* | *1847 (60)* | *308 (46.9)* | *< 0.001* | *3737* | *1595 (61)* | *560 (50)* | *< 0.001* | *3737* |
| *eGFR 30-60 (ml/min/1.73m²)* | *416 (13.5)* | *154 (23.4)* | *-* |  | *340 (13)* | *230 (20.5)* | *-* |  |
| *eGFR <30 (ml/min/1.73m²)* | *49 (1.6)* | *21 (3.2)* | *-* |  | *39 (1.5)* | *31 (2.8)* | *-* |  |
| *missing information on eGFR* | *768 (24.9)* | *174 (26.5)* | *-* |  | *642 (24.5)* | *300 (26.8)* | *-* |  |
| ***peak CK-MB (U/L)*** | *160.7 (162.4)* | *193.6 (231.1)* | *0.0033* | *3593* | *162.1 (161.4)* | *176.7 (208.4)* | *0.0239* | *3593* |
| ***peak CRP levels (mg/l)*** | *6.8 (7.9)* | *11.7 (9.6)* | *< 0.001* | *3679* | *6.6 (7.9)* | *10.2 (9.2)* | *< 0.001* | *3679* |
| ***Treatment*** | | | | | | | | |
| ***PCI*** | *2630 (85.4)* | *480 (73.1)* | *< 0.001* | *3737* | *2253 (86.1)* | *857 (76.4)* | *< 0.001* | *3737* |
| ***Bypass therapy*** | *272 (8.8)* | *85 (12.9)* | *0.0307* | *3737* | *228 (8.7)* | *129 (11.5)* | *0.0093* | *3737* |
| ***i.v. thrombolysis therapy*** | *277 (9)* | *53 (8.1)* | *0.9047* | *3737* | *239 (9.1)* | *91 (8.1)* | *0.3459* | *3737* |
| ***Any reperfusion therapy*** | *2910 (94.5)* | *567 (86.3)* | *< 0.001* | *3737* | *2489 (95.1)* | *988 (88.1)* | *< 0.001* | *3737* |
| ***Medication at discharge*** | | | | | | | | |
| ***ACE blockers*** | *2471 (81.8)* | *469 (73.5)* | *0.001* | *3660* | *2117 (82.4)* | *823 (75.4)* | *< 0.001* | *3660* |
| ***ATII antagonist*** | *242 (8)* | *52 (8.2)* | *0.9594* | *3659* | *196 (7.6)* | *98 (9)* | *0.1909* | *3659* |
| ***beta blockers*** | *2919 (96.6)* | *601 (94.1)* | *0.0035* | *3661* | *2478 (96.5)* | *1042 (95.3)* | *0.1147* | *3661* |
| ***antiplatelet drug*** | *2986 (98.8)* | *613 (95.9)* | *< 0.001* | *3661* | *2537 (98.8)* | *1062 (97.2)* | *< 0.001* | *3661* |
| ***statins*** | *2837 (93.9)* | *560 (87.8)* | *< 0.001* | *3660* | *2424 (94.4)* | *973 (89.1)* | *< 0.001* | *3660* |

***Table 2:*** *Baseline characteristics of NSTEMI patients with available data on long-term survival. Categorical data presented as total numbers (%). Numeric data is presented as mean (SD).*

| ***NSTEMI*** | ***Shock Index*** | | | | ***Modified shock index*** | | | |
| --- | --- | --- | --- | --- | --- | --- | --- | --- |
|  | ***SI ≤ 0.58 (n=4025)*** | ***SI > 0.58 (n=2412)*** | ***p-value*** | ***n*** | ***mSI ≤ 0.85 (n=4385)*** | ***mSI > 0.85 (n=2052)*** | ***p-value*** | ***n*** |
| ***sex male*** | *2987 (74.2)* | *1663 (68.9)* | *< 0.001* | *6437* | *3274 (74.7)* | *1376 (67.1)* | *< 0.001* | *6437* |
| ***age*** | *64.9 (10.6)* | *65.6 (11)* | *0.0206* | *6437* | *64.6 (10.7)* | *66.4 (10.7)* | *< 0.001* | *6437* |
| ***Number of patients who survived > 3 years (survival rate)*** | *3257 (88.6)* | *1679(75.2)* | *< 0.001* | *5908* | *3557 (88.9)* | *1379 (72.3)* | *< 0.001* | *5908* |
| ***Vital parameters*** | | | | | | | | |
| ***Heart rate*** | *71.8 (12.5)* | *96.9 (21.8)* | *< 0.001* | *6437* | *72.7 (12.9)* | *99.3 (22.3)* | *< 0.001* | *6437* |
| ***Systolic blood pressure*** | *156.5 (24.2)* | *130.5 (23.6)* | *< 0.001* | *6437* | *154.3 (24.7)* | *130.6 (24.8)* | *< 0.001* | *6437* |
| ***Diastolic blood pressure*** | *85.4 (15.3)* | *77.3 (15.8)* | *< 0.001* | *6437* | *86.3 (14.8)* | *74 (15.2)* | *< 0.001* | *6437* |
| ***SI*** | *0.5 (0.1)* | *0.8 (0.2)* | *< 0.001* | *6437* | *0.5 (0.1)* | *0.8 (0.2)* | *< 0.001* | *6437* |
| ***mSI*** | *0.7 (0.1)* | *1 (0.3)* | *< 0.001* | *6437* | *0.7 (0.1)* | *1.1 (0.3)* | *< 0.001* | *6437* |
| ***Comorbidities*** | | | | | | | | |
| ***hypertension*** | *3347 (83.2)* | *1899 (78.7)* | *< 0.001* | *6437* | *3605 (82.2)* | *1641 (80)* | *0.0337* | *6437* |
| ***diabetes*** | *1260 (31.3)* | *928 (38.5)* | *< 0.001* | *6437* | *1348 (30.7)* | *840 (40.9)* | *< 0.001* | *6437* |
| ***hyperlipidemia*** | *2653 (65.9)* | *1444 (59.9)* | *< 0.001* | *6437* | *2871 (65.5)* | *1226 (59.7)* | *< 0.001* | *6437* |
| ***Smoking status*** | | | | | | | | |
| *current smoker* | *1073 (26.7)* | *706 (29.3)* | *< 0.001* | *6437* | *1216 (27.7)* | *563 (27.4)* | *< 0.001* | *6437* |
| *never smoker* | *1355 (33.7)* | *816 (33.8)* | *-* |  | *1471 (33.5)* | *700 (34.1)* | *-* |  |
| *ex-smoker* | *1353 (33.6)* | *658 (27.3)* | *-* |  | *1443 (32.9)* | *568 (27.7)* | *-* |  |
| *no information on smoking status* | *244 (6.1)* | *232 (9.6)* | *-* |  | *255 (5.8)* | *221 (10.8)* | *-* |  |
| ***Clinical characteristics*** | | | | | | | | |
| ***typical chest-pain symptoms*** | *3390 (84.2)* | *1684 (69.8)* | *< 0.001* | *6437* | *3684 (84)* | *1390 (67.7)* | *< 0.001* | *6437* |
| ***Heart rhythm (admission ECG)*** | | | | | | | | |
| *Sinus rhythm* | *1057 (92.1)* | *566 (78.4)* | *< 0.001* | *1870* | *1135 (91.8)* | *488 (77)* | *< 0.001* | *1870* |
| *atrial fibrillation* | *74 (6.4)* | *129 (17.9)* | *-* |  | *83 (6.7)* | *120 (18.9)* | *-* |  |
| *pacemaker rhythm* | *4 (0.3)* | *1 (0.1)* | *-* |  | *4 (0.3)* | *1 (0.2)* | *-* |  |
| *ventricular tachycardia/ ventricular fibrillation* | *1 (0.1)* | *4 (0.6)* | *-* |  | *1 (0.1)* | *4 (0.6)* | *-* |  |
| *other type / unknown* | *12 (1.1)* | *22 (3.0)* | *-* |  | *13 (1.0)* | *21 (3.3)* | *-* |  |
| ***days in intensive care*** | *2.7 (4.2)* | *4.5 (7.1)* | *< 0.001* | *6169* | *2.8 (4.5)* | *4.7 (7.2)* | *< 0.001* | *6169* |
| ***any in-hospital complication*** | *398 (9.9)* | *378 (15.7)* | *< 0.001* | *6437* | *436 (9.9)* | *340 (16.6)* | *< 0.001* | *6437* |
| ***In-hospital complication: cardiogenic shock*** | *32 (0.8)* | *109 (4.5)* | *< 0.001* | *6437* | *41 (0.9)* | *100 (4.9)* | *< 0.001* | *6437* |
| ***left ventricular EF*** | | | | | | | | |
| *≤ 30%* | *87 (2.2)* | *219 (9.1)* | *< 0.001* | *6437* | *106 (2.4)* | *200 (9.7)* | *< 0.001* | *6437* |
| *> 30%* | *3061 (76)* | *1605 (66.5)* | *-* |  | *3339 (76.1)* | *1327 (64.7)* | *-* |  |
| *no information on EF* | *877 (21.8)* | *588 (24.4)* | *-* |  | *940 (21.4)* | *525 (25.6)* | *-* |  |
| ***Kidney function*** | | | | | | | | |
| *eGFR > 60 (ml/min/1.73m²)* | *2247 (55.8)* | *1041 (43.2)* | *< 0.001* | *6437* | *2452 (55.9)* | *836 (40.7)* | *< 0.001* | *6437* |
| *eGFR 30-60 (ml/min/1.73m²)* | *708 (17.6)* | *654 (27.1)* | *-* |  | *742 (16.9)* | *620 (30.2)* | *-* |  |
| *eGFR <30 (ml/min/1.73m²)* | *137 (3.4)* | *190 (7.9)* | *-* |  | *152 (3.5)* | *175 (8.5)* | *-* |  |
| *missing information on eGFR* | *933 (23.2)* | *527 (21.8)* | *-* |  | *1039 (23.7)* | *421 (20.5)* | *-* |  |
| ***peak CK-MB (U/L)*** | *60.2 (76.2)* | *70.9 (103.9)* | *< 0.001* | *5406* | *61.6 (77.4)* | *69.9 (106.6)* | *0.0012* | *5406* |
| ***peak CRP levels (mg/l)*** | *6.5 (8.3)* | *9.8 (9.6)* | *< 0.001* | *6188* | *6.5 (8.3)* | *10.2 (9.7)* | *< 0.001* | *6188* |
| ***Treatment*** | | | | | | | | |
| ***PCI*** | *2756 (68.5)* | *1290 (53.5)* | *< 0.001* | *6437* | *2991 (68.2)* | *1055 (51.4)* | *< 0.001* | *6437* |
| ***Bypass therapy*** | *656 (16.3)* | *466 (19.3)* | *0.0022* | *6437* | *728 (16.6)* | *394 (19.2)* | *0.0115* | *6437* |
| ***i.v. thrombolysis therapy*** | *63 (1.6)* | *32 (1.3)* | *0.5084* | *6437* | *72 (1.6)* | *23 (1.1)* | *0.1324* | *6437* |
| ***Any reperfusion therapy*** | *3372 (83.8)* | *1741 (72.2)* | *< 0.001* | *6437* | *3677 (83.9)* | *1436 (70)* | *< 0.001* | *6437* |
| ***Medication at discharge*** | | | | | | | | |
| ***ACE blockers*** | *2882 (73.7)* | *1681 (72.3)* | *0.2325* | *6237* | *3150 (73.9)* | *1413 (71.6)* | *0.0594* | *6237* |
| ***ATII antagonist*** | *435 (11.1)* | *214 (9.2)* | *0.0183* | *6235* | *459 (10.8)* | *190 (9.6)* | *0.1849* | *6235* |
| ***beta blockers*** | *3657 (93.5)* | *2175 (93.5)* | *0.996* | *6238* | *3998 (93.8)* | *1834 (92.9)* | *0.2238* | *6238* |
| ***antiplatelet drug*** | *3834 (98.0)* | *2193 (94.2)* | *< 0.001* | *6238* | *4169 (97.8)* | *1858 (94.1)* | *< 0.001* | *6238* |
| ***statins*** | *3542 (90.6)* | *1988 (85.4)* | *< 0.001* | *6237* | *3857 (90.5)* | *1673 (84.8)* | *< 0.001* | *6237* |

***Table 3:*** *COX regression models with time-split function for shock index. The low shock index group was set as the reference group.*

| ***SI group*** | ***Total Sample*** | | ***STEMI*** | | ***NSTEMI*** | |
| --- | --- | --- | --- | --- | --- | --- |
|  | *HR [95% CI]* | *p-value* | *HR [95% CI]* | *p-value* | *HR [95% CI]* | *p-value* |
| ***Adjusted for sex and age*** | | | | | | |
| ***High SI***  ***< 1 year*** | 2.63 [ 2.23 - 3.1 ] | < 0.001 | 2.93 [ 2.09 - 4.12 ] | < 0.001 | 2.57 [ 2.13 - 3.1 ] | < 0.001 |
| ***High SI***  ***1 - 3 years*** | 2.03 [ 1.74 - 2.35 ] | < 0.001 | 1.71 [ 1.27 - 2.3 ] | < 0.001 | 2.19 [ 1.84 - 2.6 ] | < 0.001 |
| ***High SI***  ***3-6 years*** | 1.84 [ 1.61 - 2.12 ] | < 0.001 | 1.74 [ 1.34 - 2.24 ] | < 0.001 | 1.92 [ 1.63 - 2.26 ] | < 0.001 |
| ***High SI***  ***6-9 years*** | 1.75 [ 1.49 - 2.05 ] | < 0.001 | 1.24 [ 0.94 - 1.63 ] | 0.125 | 2.12 [ 1.73 - 2.58 ] | < 0.001 |
| ***High SI***  ***9 - 12 years*** | 1.58 [ 1.29 - 1.93 ] | < 0.001 | 1.17 [ 0.82 - 1.66 ] | 0.3865 | 1.89 [ 1.48 - 2.42 ] | < 0.001 |
| ***High SI***  ***> 12 years*** | 1.2 [ 0.98 - 1.48 ] | 0.0758 | 1.02 [ 0.74 - 1.41 ] | 0.8941 | 1.39 [ 1.06 - 1.81 ] | 0.0171 |
| ***Fully adjusted model**** | | | | | | |
| ***High SI***  ***< 1 year*** | 1.62 [ 1.36 - 1.93 ] | < 0.001 | 1.87 [ 1.3 - 2.69 ] | 0.001 | 1.54 [ 1.27 - 1.88 ] | < 0.001 |
| ***High SI***  ***1 - 3 years*** | 1.44 [ 1.23 - 1.69 ] | < 0.001 | 1.26 [ 0.92 - 1.73 ] | 0.1581 | 1.53 [ 1.27 - 1.83 ] | < 0.001 |
| ***High SI***  ***3-6 years*** | 1.39 [ 1.21 - 1.61 ] | < 0.001 | 1.41 [ 1.08 - 1.85 ] | 0.0141 | 1.4 [ 1.18 - 1.66 ] | < 0.001 |
| ***High SI***  ***6-9 years*** | 1.45 [ 1.23 - 1.72 ] | < 0.001 | 1.06 [ 0.8 - 1.42 ] | 0.7084 | 1.73 [ 1.41 - 2.14 ] | < 0.001 |
| ***High SI***  ***9 - 12 years*** | 1.41 [ 1.15 - 1.74 ] | 0.0013 | 1.16 [ 0.81 - 1.66 ] | 0.4087 | 1.58 [ 1.23 - 2.03 ] | < 0.001 |
| ***High SI***  ***> 12 years*** | 1.16 [ 0.94 - 1.43 ] | 0.1676 | 0.97 [ 0.69 - 1.36 ] | 0.8473 | 1.34 [ 1.02 - 1.76 ] | 0.0365 |

* adjusted for sex, age, typical chest pain symptoms, diabetes, smoking, hyperlipidemia, hypertension, left-ventricular EF ≤ 30%, impaired renal function (according to GFR), any in-hospital complication, PCI, Bypass surgery and Lysis therapy.

***Table 4:*** *COX regression models with time-split function for modified shock index. The low modified shock index group was set as the reference group.*

| ***mSI group*** | ***Total Sample*** | | ***STEMI*** | | ***NSTEMI*** | |
| --- | --- | --- | --- | --- | --- | --- |
|  | *HR [95% CI]* | *p-value* | *HR [95% CI]* | *p-value* | *HR [95% CI]* | *p-value* |
| ***Adjusted for sex and age*** | | | | | | |
| ***High mSI***  ***< 1 year*** | 2.81 [ 2.39 - 3.3 ] | < 0.001 | 2.75 [ 1.99 - 3.82 ] | < 0.001 | 2.84 [ 2.35 - 3.42 ] | < 0.001 |
| ***High mSI***  ***1 - 3 years*** | 2.16 [ 1.86 - 2.51 ] | < 0.001 | 1.71 [ 1.27 - 2.31 ] | < 0.001 | 2.37 [ 1.99 - 2.82 ] | < 0.001 |
| ***High mSI***  ***3-6 years*** | 2.08 [ 1.81 - 2.38 ] | < 0.001 | 1.89 [ 1.46 - 2.45 ] | < 0.001 | 2.19 [ 1.86 - 2.58 ] | < 0.001 |
| ***High mSI***  ***6-9 years*** | 1.75 [ 1.49 - 2.07 ] | < 0.001 | 1.08 [ 0.81 - 1.45 ] | 0.584 | 2.28 [ 1.86 - 2.79 ] | < 0.001 |
| ***High mSI***  ***9 - 12 years*** | 1.52 [ 1.23 - 1.88 ] | < 0.001 | 1.19 [ 0.83 - 1.72 ] | 0.3489 | 1.79 [ 1.38 - 2.31 ] | < 0.001 |
| ***High mSI***  ***> 12 years*** | 1.26 [ 1.02 - 1.56 ] | 0.0362 | 1.16 [ 0.83 - 1.62 ] | 0.3812 | 1.39 [ 1.04 - 1.85 ] | 0.0269 |
| ***Fully adjusted model**** | | | | | | |
| ***High mSI***  ***< 1 year*** | 1.68 [ 1.41 - 1.99 ] | < 0.001 | 1.73 [ 1.22 - 2.46 ] | 0.003 | 1.66 [ 1.36 - 2.02 ] | < 0.001 |
| ***High mSI***  ***1 - 3 years*** | 1.48 [ 1.27 - 1.73 ] | < 0.001 | 1.23 [ 0.9 - 1.7 ] | 0.2179 | 1.60 [ 1.33 - 1.92 ] | < 0.001 |
| ***High mSI***  ***3-6 years*** | 1.53 [ 1.32 - 1.77 ] | < 0.001 | 1.55 [ 1.18 - 2.04 ] | 0.0021 | 1.55 [ 1.3 - 1.84 ] | < 0.001 |
| ***High mSI***  ***6-9 years*** | 1.44 [ 1.21 - 1.71 ] | < 0.001 | 0.92 [ 0.68 - 1.25 ] | 0.528 | 1.84 [ 1.49 - 2.28 ] | < 0.001 |
| ***High mSI***  ***9 - 12 years*** | 1.34 [ 1.08 - 1.66 ] | 0.0128 | 1.23 [ 0.84 - 1.8 ] | 0.3622 | 1.46 [ 1.12 - 1.9 ] | 0.0063 |
| ***High mSI***  ***> 12 years*** | 1.19 [ 0.95 - 1.48 ] | 0.1484 | 1.12 [ 0.78 - 1.59 ] | 0.5835 | 1.28 [ 0.95 - 1.73 ] | 0.1082 |

* adjusted for sex, age, typical chest pain symptoms, diabetes, smoking, hyperlipidemia, hypertension, left-ventricular EF≤ 30%, impaired renal function (according to GFR), any in-hospital complication, PCI, Bypass surgery and Lysis therapy.

***Figure 1:*** *ROC curves for 3-year mortality including all recorded cases by the registry (patients who died within 28 days after AMI included). P-values are calculated by comparing the AUC between SI and mSI using bootstrapping.*


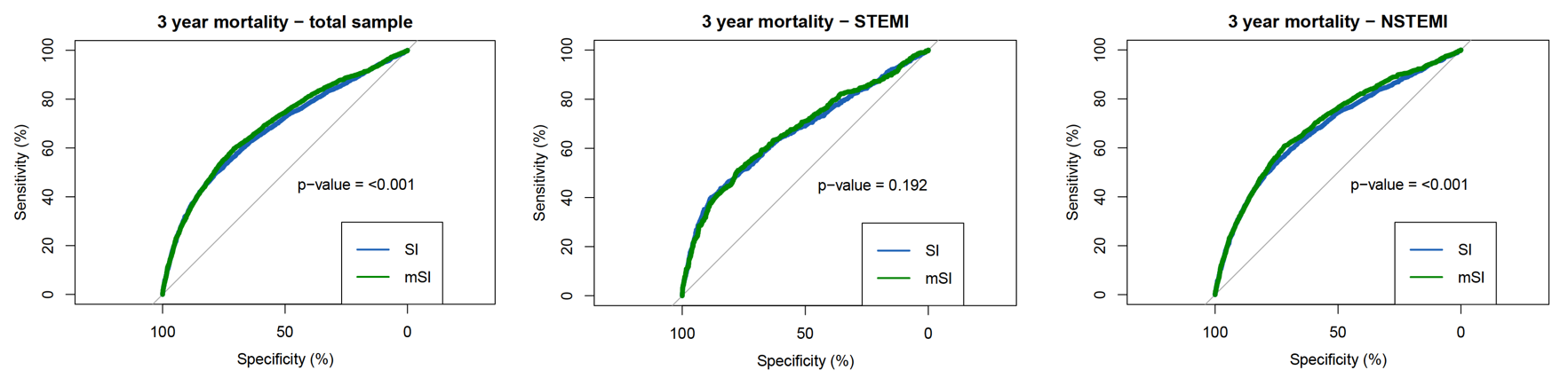


***Table 4:*** *AUC values for 3-year mortality including all recorded cases by the registry (patients who died within 28 days after AMI included)*

|  | AUC (95%CI) | | p-value |
| --- | --- | --- | --- |
|  | SI | mSI |  |
| Total sample | 0.6714 (0.6587-0.6843) | 0.6856 (0.673-0.6988) | <0.001 |
| STEMI | 0.6609 (0.6356-0.6885) | 0.6673 (0.6411-0.6922) | 0.192 |
| NSTEMI | 0.6778 (0.6623-0.6923) | 0.6934 (0.6791-0.708) | <0.001 |

***Figure 2:*** *ROC curves for 28 day mortality including all recorded cases by the registry. P-values are calculated by comparing the AUC between SI and mSI using bootstrapping.*


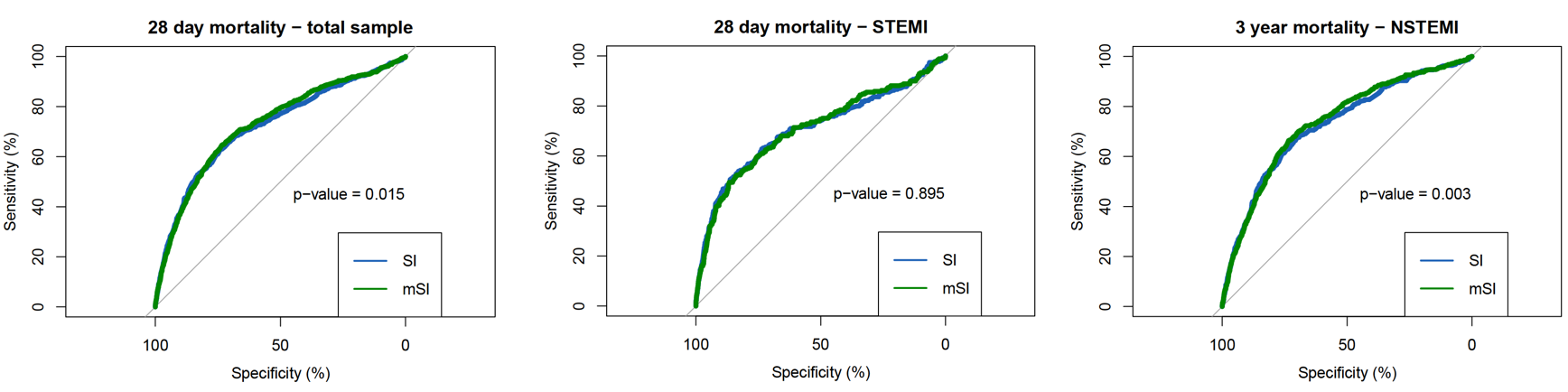


***Table 5:*** *AUC values for 28 day mortality including all recorded cases by the registry (patients who died within 28 days after AMI included)*

|  | AUC (95%CI) | | p-value |
| --- | --- | --- | --- |
|  | SI | mSI |  |
| Total sample | 0.7209 (0.7006-0.7414) | 0.7283 (0.7086-0.7475) | 0.015 |
| STEMI | 0.7066 (.6662-0.7452) | 0.7074 (0.6702-0.7436) | 0.895 |
| NSTEMI | 0.7282 (0.704-0.7506) | 0.7381 (0.7155-0.7606) | 0.003 |

***Table 6:*** *Display of survival rates: 28 day survival, 3 year survival including all patients, 3 year survival of patients who survived the first 28 days*

|  | ***SI*** | | ***mSI*** | |
| --- | --- | --- | --- | --- |
|  | SI ≤ 0.58 | SI > 0.58 | mSI ≤ 0.85 | mSI > 0.85 |
| ***28 day survival rate*** | 96.8 | 88.4 | 96.9 | 86.8 |
| ***3 year survival rate***  ***(all cases included)*** | 86.6 | 68.1 | 86.8 | 64.9 |
| ***3 year survival rate***  ***(28day - 3 years)*** | 90.3 | 79.6 | 90.4 | 77.2 |
